# Comparison of Different Procedures in a Combination of Ab Interno Microhook Trabeculotomy and Cataract Surgery

**DOI:** 10.3390/jcm11030738

**Published:** 2022-01-29

**Authors:** Yusaku Miura, Ken Fukuda

**Affiliations:** Department of Ophthalmology and Visual Science, Kochi Medical School, Kochi University, Nankoku 783-8505, Japan; miurasaku@kochi-u.ac.jp

**Keywords:** minimally invasive glaucoma surgery (MIGS), microhook, hyphema, surgical efficacy, surgical complication

## Abstract

The purpose of this study was to compare the clinical outcomes of ab interno microhook trabeculotomy (µLOT) before and after cataract surgery for the combination of µLOT and cataract surgery. This retrospective case series included 40 eyes that underwent µLOT combined with cataract surgery at Kochi University Hospital. Groups 1 (20 eyes) and 2 (20 eyes) included eyes that underwent µLOT before and after cataract surgery, respectively. The patient characteristics and clinical outcomes were also analyzed. The mean preoperative intraocular pressure (IOP) in Groups 1 and 2 (26.1 ± 12.2 mmHg and 20.6 ± 8.8 mmHg) was reduced significantly to 14.1 ± 3.3 mmHg and 12.9 ± 3.2 mmHg, respectively, at 5–7 months postoperatively. The median preoperative number of antiglaucoma medications in Groups 1 and 2 (4.0 and 3.5) also decreased significantly, to 2.5 and 1.0, respectively, at 5–7 months postoperatively. Postoperative hyphema with niveau formation in Groups 1 and 2 was observed in one eye (5.0%) and six eyes (30.0%), respectively. For the combination of µLOT and cataract surgery, performing µLOT before cataract surgery may be less likely to result in postoperative hyphema with niveau formation.

## 1. Introduction

Trabeculotomy (LOT) is performed in patients with mild to moderate glaucoma other than angle-closure glaucoma to reduce the intraocular pressure (IOP) by reducing the resistance to aqueous flow by incising the trabecular meshwork and the inner wall of the Schlemm canal [1,2,3,4]. Recently, minimally invasive glaucoma surgery (MIGS) has been reported as a safer and less traumatic ab interno surgical procedure [5,6,7,8]. Unlike conventional ab externo LOT using metal trabeculotomy, MIGS tends to involve minimal trauma with very little or no scleral dissection, minimal or no conjunctival manipulation, good safety, and rapid recovery [9]. Among MIGS, the microhook LOT (µLOT) reported by Tanito is especially easy and less time-consuming to perform because the tip of the hook is much smaller than that in the other gonio-surgery devices, and the intracameral manipulation of the hook is easier than in the other devices [10]. A recent study has revealed that µLOT is effective in reducing IOP especially in glaucoma patients with older age, steroid-induced glaucoma, or developmental glaucoma [11]. Another advantage of µLOT is that it does not require expensive devices [10]. However, a study of combined cataract surgery and µLOT reported postoperative hyphema with niveau formation in 41% of cases [12], while another study of combined cataract surgery and Kahook dual blade categorized as MIGS reported no postoperative hyphema with niveau formation [13]. These studies utilized different surgical procedures and instruments. Hirabayashi et al. performed ab interno LOT using a Kahook dual blade before cataract surgery [13]. In contrast, Tanito et al. performed µLOT after cataract surgery; however, the reason for performing µLOT after and not before cataract surgery was not described [12]. Thus, it is not clear whether µLOT should be performed before or after cataract surgery. We hypothesized that the frequency of hyphema may vary depending on whether μLOT is performed before or after cataract surgery. Therefore, the present study compared µLOT before and after cataract surgery to determine if there were differences in clinical outcomes according to the timing of the cataract procedure.

## 2. Materials and Methods

### 2.1. Methods

Our study included patients with glaucoma who underwent µLOT before (Group 1) or after (Group 2) cataract surgery.

Most surgeries were performed by a single surgeon (Y.M.). Some surgeries were performed by four doctors supervised by Y.M. between April 2019 and June 2021 at Kochi University Hospital, Japan. We selected patients with early stage glaucoma who had not yet undergone surgery. All eyes included in this study had an open-angle and identifiable trabecular meshwork under gonioscopy. We excluded patients with angle-closure glaucoma, a postoperative follow-up of <5 months, or a history of glaucoma surgery. Data including age, sex, glaucoma type, IOP based on Goldmann applanation tonometry, number of antiglaucoma medications, best-corrected visual acuity (BCVA), surgical time, intraoperative and postoperative complications, and interventions for complications were collected from the medical charts.

### 2.2. Surgical Procedure

**Figure 1:**
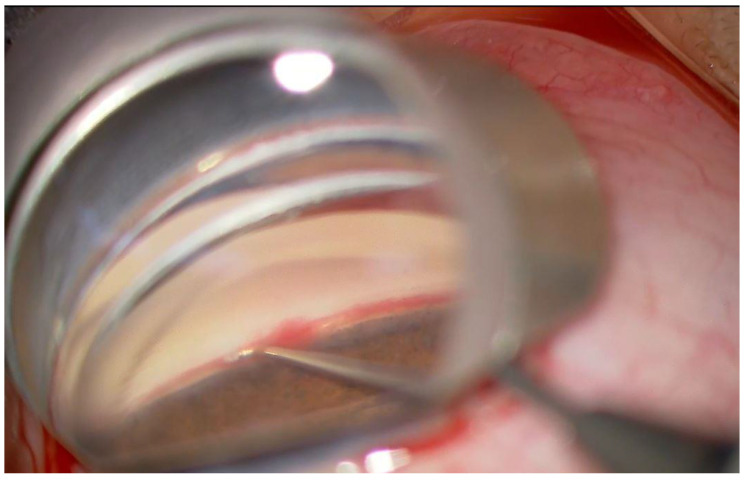
Intraoperative photograph of microhook ab interno trabeculotomy. Observation of the anterior-chamber angle using a Hill surgical gonioprism shows the trabecular meshwork and the process of cutting through the inner wall of Schlemm’s canal circumferentially using the microhook inserted through a small corneal incision.

First, peribulbar anesthesia was performed in all eyes using a sub-Tenon injection of 2% lidocaine. In Group 1, µLOT was performed using the following procedure: viscoelastic material (1% sodium hyaluronate, Opegan Hi, Santen Pharmaceutical, Osaka, Japan) was injected into the anterior chamber (AC) through two corneal ports on the temporal side created using a 20-gauge microvitreoretinal (MVR) knife (Mani, Utsunomiya, Japan). Using a Hill surgical gonioprism (Ocular Instruments, Bellevue, WA, USA) to observe the angle opposite the corneal port, a microhook was inserted into the AC through the corneal port. The tip of the microhook was then inserted into the Schlemm’s canal and moved circumferentially to incise the inner wall of the Schlemm’s canal and trabecular meshwork over 2 clock hours (Figure 1). Using the same procedure, LOT was performed using a microhook inserted through another corneal port. That is, a trabecular meshwork totaling 120 degrees or more was incised. In Group 1, standard phacoemulsification with IOL implantation was performed through an additional temporal corneal 2.8 mm incision. In Group 2, µLOT was performed using the same procedure as described above after standard phacoemulsification with IOL implantation. After surgery, postoperative long-standing hyphema was treated with anterior chamber washout. Postoperative blood accumulation in the lens bag was treated using Nd:YAG laser capsulotomy to disperse the accumulated blood.

### 2.3. Outcome Measures

The primary outcome was the frequency of hyphema with niveau formation, which was defined as a hemorrhagic niveau of 0.5 mm or more at the inferior part of the anterior chamber. The secondary outcome was IOP measured 5–7 months after surgery. Surgical success was defined as: (1) 6 ≤ IOP ≤ 21 and a reduction of more than 20% with or without antiglaucoma medications; (2) no loss of light perception; and (3) no additional glaucoma surgery. Transient IOP elevation within 1 month postoperatively was considered an IOP spike and was not classified as a surgical failure.

### 2.4. Statistical Analysis

BCVA was converted to the logarithm of the minimum angle of resolution (logMAR) for statistical analysis. Wilcoxon signed-rank tests were used to compare IOP, number of antiglaucoma medications, and BCVA values preoperatively and 5–7 months postoperatively. Wilcoxon rank-sum tests were used to evaluate the group differences between continuous variables. The incidence of complications was compared between the groups using Fisher exact tests. Statistical significance was set at *p* < 0.05. All data were entered into an Excel spreadsheet (Microsoft Corp., Redmond, WA, USA) and analyzed using Excel 2016 with the add-in software Statcel 4.

## 3. Results

This retrospective study included a total of 40 eyes from 31 patients who underwent surgery. Groups 1 and 2 comprised 20 eyes of 16 patients and 20 eyes of 15 patients, respectively. The patient characteristics are shown in Table 1. The two groups were well-matched for demographic characteristics.

The mean IOP in Group 1 decreased significantly, from 26.1 ± 12.2 mmHg at baseline to 14.1 ± 3.3 mmHg at 5–7 months postoperatively (*p* = 0.000089) (Table 2). Similarly, the mean IOP in Group 2 decreased significantly, from 20.6 ± 8.8 mmHg at baseline to 12.9 ± 3.2 mmHg at 5–7 months postoperatively (*p* = 0.00014). The mean (±SD) percentage of IOP reduction from baseline was 38.3% (±20.0) and 31.3% (±21.4) in Groups 1 and 2, respectively. The mean IOPs did not differ significantly between the two groups at baseline and 5–7 months postoperatively. The median number of antiglaucoma medications in Group 1 decreased significantly from 4.0 (3–5) at baseline to 2.5 (2–3) at 5–7 months postoperatively (*p* = 0.0011). Similarly, the median number of antiglaucoma medications in Group 2 also decreased significantly, from 3.5 (2–4.25) to 1.0 (0–3) (*p* = 0.00048). The median number of antiglaucoma medications did not differ significantly between the two groups at baseline and 5–7 months postoperatively. The mean best corrected visual acuity (BCVA) in Group 1 improved significantly from 0.27 ± 0.28 at baseline to −0.004 ± 0.18 at 5–7 months postoperatively (*p* = 0.00032). Similarly, the mean BCVA improved significantly from 0.21 ± 0.45 to 0.058 ± 0.38 in Group 2 (*p* = 0.0016). The mean BCVA did not differ significantly between the two groups at baseline and 5–7 months postoperatively. The mean surgical times for Groups 1 and 2 were 17.3 ± 5.7 min and 20.9 ± 6.9 min, respectively.

**Figure 2:**
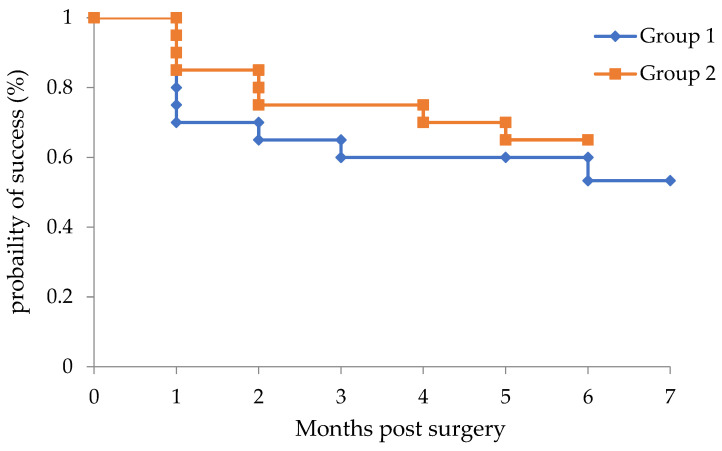
Kaplan–Meier survival curves showing the success rate of IOP control after surgery. Surgical success was defined as: (1) 6 ≤ IOP ≤ 21, and a reduction by more than 20% with or without antiglaucoma medications; (2) no loss of light perception; (3) no additional glaucoma surgery. Transient IOP elevation within 1 month postoperatively was considered an IOP spike and not classified as surgical failure.

Figure 2 shows the Kaplan–Meier survival curves used to compare the surgical outcomes between Groups 1 and 2. The success rates in Groups 1 and 2 were 53.3% and 65.0%, respectively (*p* = 0.54).

Table 3 lists the complications and subsequent interventions. All cases showed intraoperative blood reflux into the anterior chamber from the incised angle. Postoperative hyphema with niveau formation in Groups 1 and 2 was observed in one eye and six eyes, respectively. In five of these eyes, the hyphema resolved spontaneously within 1 week postoperatively without any intervention. However, we performed hyphema washout about a week after the surgery in two eyes of Group 2 that showed no tendency to improve. The patients who had taken any anticoagulants were two of Group 2, but they did not show postoperative hyphema with niveau formation. Postoperative transient IOP elevation was observed in three eyes in Group 1 and four eyes in Group 2, all of which normalized within 1 week following the administration of glaucoma medications. Postoperative blood accumulation in the lens bag was observed in one eye in Group 2, which was treated with Nd:YAG laser capsulotomy to spread the accumulated blood.

## 4. Discussion

Generally, trabeculectomy remains the gold standard of glaucoma surgery that can significantly reduce IOP. However, trabeculectomy can result in severe postoperative complications, such as hypotony maculopathy, choroidal hemorrhage, bleb infections, and endophthalmitis. In contrast, LOT is less likely to induce postoperative vision-threatening complications after trabeculectomy, but postoperative hyphema is likely to occur [1,14].

In our study, all cases showed intraoperative blood reflux from the incised angle into the anterior chamber. This intraoperative hemorrhage is common in µLOT and other MIGS procedures [12,15,16]. Blood reflux occurs when unroofing Schlemm’s canal in the setting of intraoperative hypotony and should be considered an expected event in these surgical procedures. The incidence of postoperative hyphema with niveau formation in Group 2 was 30.0%, which was similar to that reported in a previous study of combined cataract surgery and µLOT [12]. In a previous study of µLOT only [15], postoperative hyphema with niveau formation occurred in 38% of cases, a rate similar to those in a previous study [12] and to that of Group 2 in the present study. Gonioscopy-assisted transluminal trabeculotomy (GATT) is another ab interno trabeculotomy similar to µLOT [17,18]. The common postoperative complication of GATT is hyphema, and the rate of hyphema is reportedly approximately 30% [19,20]. Hyphema is the most common postoperative complication of all types of LOT. A recent study has also revealed that 31% of 560 eyes that underwent μLOT alone or μLOT combined with cataract surgery had hyphema with niveau formation [11]. However, the incidence of postoperative hyphema with niveau formation in Group 1 was 5.0%, significantly lower than the incidence in Group 2. This is because perfusion pressure during cataract surgery may help prevent postoperative hyphema. In Group 1, perfusion pressure during cataract surgery after µLOT increased the intraoperative IOP and compressed the incised wound. As a result, intraoperative blood reflux was reduced, and postoperative hyphema was less likely to occur. In contrast, in Group 2 and previous studies [12,15], µLOT was performed after or without cataract surgery. Therefore, a reduction in intraoperative blood reflux due to perfusion pressure could not be expected. That is, to avoid postoperative hyphema with niveau formation, µLOT should be performed before rather than after cataract surgery when µLOT and cataract surgery are combined.

The procedures in Groups 1 and 2 have advantages. The advantages of performing µLOT before cataract surgery include obtaining a good gonioscopic view through a clear cornea and avoiding anterior chamber instability from the large keratome incision used in cataract surgery. In contrast, the advantages of performing µLOT after cataract surgery include obtaining good visibility of the trabecular meshwork in the open angle due to cataract removal. However, regardless of the procedure used, µLOT combined with cataract surgery was completed successfully in our study because the ease of performing this surgery was not affected. We observed no significant difference in surgical times between Groups 1 and 2 (*p* = 0.13). The IOP and number of antiglaucoma medications preoperatively and 5–7 months postoperatively did not differ significantly between Groups 1 and 2. Tanito et al. reported that µLOT combined with cataract surgery decreased the mean IOP and number of antiglaucoma medications from 16.4 mmHg and 2.4 preoperatively to 11.8 mmHg and 2.1 at the final 9.5-month evaluation [12], similar to the outcomes in Groups 1 and 2 in the present study.

The limitations of our study are the small sample size, the non-randomized nature of the study, and the short follow-up period. To clarify the differences in clinical outcomes of µLOT before and after cataract surgery, a larger number of cases and longer follow-up periods are needed. Furthermore, randomized studies should be performed to eliminate biases such as patient backgrounds.

In conclusion, the results of our study showed that both µLOT before and after cataract surgery effectively reduced the IOP and number of antiglaucoma medications. However, the incidence of postoperative hyphema with niveau formation was lower for µLOT before rather than after cataract surgery. Thus, µLOT may be better performed before cataract surgery when performing combined µLOT and cataract surgery.

## Figures and Tables

**Table 1:** Patient characteristics.

	Group 1	Group 2	*p*
Number of Eyes	20	20	
Mean age (range), years	72.7 ± 9.4 (51–85)	74.2 ± 6.5 (60–82)	0.73
Sex			
Male	9 (45.0%)	5 (25.0%)	
Female	11 (55.0%)	15 (75.0%)	
Eye			
Right	7 (35.0%)	8 (40.0%)	
Left	13 (65.0%)	12 (60.0%)	
Glaucoma Type			
POAG	9 (45.0%)	9 (45.0%)	
EXG	3 (15.0%)	6 (30.0%)	
SIG	3 (15.0%)	2 (10.0%)	
UG	3 (15.0%)	2 (10.0%)	
Other	2 (10.0%)	1 (5.0%)	
Visual field mean deviation (dB)	−7.6 ± 6.4	−7.2 ± 7.3	0.57
Follow-up (months)	6.0 ± 0.73	5.6 ± 0.75	0.13

POAG, primary open angle glaucoma. EXG, exfoliation glaucoma. SIG, steroid induced glaucoma. UG, uveitis glaucoma.

**Table 2:** Surgical results.

	Group 1	Group 2	*p*
IOP (mmHg)			
Preoperative	26.1± 12.3	20.6 ± 8.8	0.13
Postoperative	14.1 ± 3.3	12.9 ± 3.2	0.33
Medication			
Preoperative	4.0 (3–5)	3.5 (2–4.25)	0.11
Postoperative	2.5 (2–3)	1.0 (0–3)	0.06
BCVA (logMAR)			
Preoperative	0.28 ± 0.28	0.21 ± 0.45	0.13
Postoperative	−0.040 ± 0.18	0.058 ± 0.38	0.55
Surgical time (min)	17.3 ± 5.7	20.9 ± 6.9	0.13

IOP, intraocular pressure; BCVA, best-corrected visual acuity; MAR, the minimum angle of resolution.

**Table 3:** Complications and interventions.

	Group 1	Group 2	*p*
Intraoperative complications			
Blood reflux	20 (100%)	20 (100%)	
Postoperative complications			
Hyphema with niveau formation	1 (5.0%)	6 (30.0%)	0.046
Transient IOP elevation (>30 mmHg)	3 (15.0%)	4 (20.0%)	0.50
Blood accumulation in the lens bag	0	1 (5.0%)	0.50
Postoperative intervention			
Hyphema washout	0	2 (10.0%)	0.24
Nd:YAG laser capsulotomy	0	1 (5.0%)	0.50

## Data Availability

Not applicable.
